# Germline BRCA1/BRCA2 mutations among high risk breast cancer patients in Jordan

**DOI:** 10.1186/s12885-018-4079-1

**Published:** 2018-02-06

**Authors:** Hikmat Abdel-Razeq, Amal Al-Omari, Farah Zahran, Banu Arun

**Affiliations:** 1 Department of Internal Medicine, King Hussein Cancer Center and University of Jordan, School of Medicine, 202 Queen Rania Al-Abdulla St., P.O. Box 1269 Al-Jubeiha, Amman, 11941 Jordan; 2 Office of Scientific Affairs and Research, King Hussein Cancer Center, 202 Queen Rania Al-Abdulla St., P.O. Box 1269 Al-Jubeiha, Amman, 11941 Jordan; 3 Department of Breast Medical Oncology, University of Texas, M D Anderson Cancer Center, 1515 Holcombe Boulevard, Houston, TX 77030 USA

**Keywords:** Breast cancer, BRCA1, BRCA2, Jordan, Hereditary breast cancer

## Abstract

**Background:**

Breast cancer is the most common malignancy and the leading cause of cancer-related deaths among Jordanian women. With a median age of 50 years at diagnosis, a higher prevalence of hereditary breast cancer may be expected. The objective of this pilot study is to evaluate, for the first time, the contribution of germline mutations in BRCA1/2 to breast cancer among Jordanian patients.

**Methods:**

Jordanian breast cancer women with a selected high risk profile were invited to participate. Peripheral blood samples were obtained for DNA extraction. A detailed 3-generation family history was also collected. BRCA sequencing was performed at a reference laboratory. Mutations were classified as deleterious, suspected deleterious, variant of uncertain significance or favor polymorphisms. Patients’ medical records were reviewed for extraction of clinical and tumor pathology data.

**Results:**

One hundred patients were enrolled to the study. Median age was 40 (22–75) years. In total, 20 patients had deleterious and 7 suspected deleterious mutations in BRCA1 or BRCA2 genes. Seven variants of uncertain significance were also detected. After excluding patients tested subsequent to the index case in their families, highest mutation rates were observed among triple negatives (9/16, 56.3%) especially among those with positive family history of breast and/or ovarian cancer (9/13, 69.2%), patients with bilateral or second primary breast cancer (10/15, 66.7%) and those with family history of male breast cancer (2/5, 40.0%).

**Conclusions:**

BRCA1/2 mutations are not uncommon among selected Jordanian females with breast cancer. The contribution of these findings to much younger age at diagnosis is debatable.

Although small, our selected patient cohort shows an important incidence of deleterious and suspected deleterious BRCA1/2 mutations suggesting that genetic testing should be offered to patients with certain high risk features.

## Background

Breast cancer is the most common cancer and the leading cause of cancer-related deaths among Jordanian women. The latest annual report of the Jordan Cancer Registry stated a total of 1067 breast cancer cases, accounting for 19.7% of all cancer cases diagnosed in Jordan [1].

Like many neighboring countries, breast cancer in Jordan presents with many peculiar features. The median age at presentation is 50 years; 10 years younger than western societies. Additionally more than a third of patients present with locally-advanced or metastatic disease highlighting the importance of early detection programs [2, 3].

Given the limited resources and recent debates about the value of national screening mammography [4–6], identifying higher risk group(s) of patients to which preventive and early detection efforts can be directed is extremely important.

Hereditary breast cancer is well-described; around 5–10% of breast cancer patients carry high risk gene mutations like BRCA1 and BRCA2 [7, 8]. Given the high penetrance rates among such mutation carriers [9, 10], it will be important to identify those patients to whom many additional risk-reduction clinical interventions, like bilateral mastectomies and oophorectomies can be performed. The Frequency of BRCA1 and BRCA2 carrier rates varies from 1/400 in the general Caucasian population to as high as 1/40 among the Ashkenazi Jewish population [11].

Data related to hereditary breast cancer among the Arab countries is very scarce; none reported from Jordan. In a recent study that included 250 high risk Lebanese patients, 14 (5.6%) were found to carry a deleterious BRCA mutation (7 BRCA1, 7 BRCA2) and 31 others (12.4%) carried a variant of uncertain significance (VUS) [12]. High risk patients were defined as those diagnosed at young age (≤40 years), those ≤50 years old with positive family history of breast or ovarian cancer and those with personal history of ovarian cancer. However, an earlier study from the same country that included 72 unrelated patients with positive family history of breast and/or ovarian cancers or with an early onset breast cancer reported higher carrier rates; deleterious BRCA1 and BRCA2 mutations were reported in 12.5% [13].

BRCA1 gene analysis was also performed in 121 Moroccan women diagnosed with breast cancer; 31.6% (6/19) of familial and 1% (1/102) of early-onset sporadic cases (< 45 years) were found to be associated with BRCA1 mutations [14]. In Egypt, 60 breast cancer patients, derived from 60 families, were selected for molecular genetic testing of BRCA1 and BRCA2 genes. The study also included 120 healthy first degree female relatives of the patients, either sisters and/or daughters, for early detection of presymptomatic breast cancer mutation carriers. Mutations were detected in 86.7% of the families; 60% were BRCA1, while 26.7% were attributable to BRCA2 mutations [15]. Few other smaller regional studies had reported variable rates [16–18]. The variability of results from the above-mentioned studies might be related to patient selection criteria, referral patterns, small number of patients enrolled and different methods of testing.

The aim of our study is to evaluate, and for the first time, the contribution of germline mutations in BRCA1/2 to breast cancer among Jordanian patients with a selected high risk profile.

## Methods

### Patient population

Jordanian breast cancer patients with a selected high risk profile; as per the National Comprehensive Cancer Network (NCCN) guidelines [19] were invited to participate. This includes patients 40 years or younger, triple negative patients (i.e. negative for estrogen receptors ER, progesterone receptors PR, and HER2 receptors) age ≤ 50 years, patients diagnosed at any age with ≥2 close relatives (any age) with breast, epithelial ovarian, fallopian tube or primary peritoneal cancer, patients with family history of male breast cancer and patients with two breast cancer primaries, or breast and ovarian/fallopian tube/primary peritoneal cancer. Eligible patients were identified by review of the King Hussein Cancer Center Tumor Registry and medical records, and approached during routine clinic visits. Patients were interviewed for 30 min for proper consent and were given full autonomy to decide whether they want to know their test result, want to inform their treating physician or place a copy of the test result in their medical record. A detailed 3-generation family history was also obtained by one of the investigators.

Patients were made aware of all clinical and psychosocial consequences of positive test results. When needed and requested by the patient, such meeting and discussion were also carried out with the spouse and/or family members.

Patients’ medical records were reviewed for extraction of clinical data and tumor pathology.

The study was funded by a competitive grant from the King Hussein Cancer Center/MD Anderson Cancer Center Sister Institution Network Fund (SINF). The study was approved by King Hussein Cancer Center Institutional Review Board (IRB) under project number 11KHCC63. All patients signed informed consent.

### BRCA1/2 testing

BRCA1/2 testing was done at no-cost to participants. Ten mL peripheral blood samples were obtained for DNA extraction. BRCA sequencing was performed at Myriad Genetics laboratory (Myriad Genetics, Salt Lake City, UT) utilizing the Comprehensive BRACAnalysis® and BRACAnalysis® Rearrangement Test (BART). Analysis consists of sequencing of all translated exons and immediately adjacent intronic regions of the BRCA1 and BRCA2 genes and a comprehensive rearrangement test of both BRCA1 and BRCA2 by quantitative PCR analysis.

A disease-causing mutation, also called deleterious mutation, pathogenic variant, predisposing mutation, and susceptibility gene, is a genetic alteration that increases an individual’s susceptibility or predisposition to a certain disease or disorder. When such a variant (or mutation) is inherited, development of symptoms is more likely, but not certain. BRCA mutations were classified as deleterious, suspected deleterious, variant of uncertain significance (VUS) or favor polymorphism based on established criteria [20].

### Statistical analysis

Patient characteristics were tabulated and described by their medians, ranges or percentages (%). Relatives tested later to the index case in the family were excluded from subsequent analyses. χ2 test or Fisher exact test were used to compare the proportion of positive BRCA1/2 deleterious/suspected deleterious mutations according to age (cut-off ≤40), triple negative status, first and/or second-degree family history of breast and/or ovarian cancer, number of first and/or second-degree relatives with breast and/or ovarian cancer (cut-off ≥2), bilateral or second primary breast cancer and family history of male breast cancer. Multivariate analysis using a logistic regression model adjusting for age, triple negative status, number of first and/or second-degree relatives with breast and/or ovarian cancer and bilateral or second primary breast cancer was performed. Odds ratios and their related 95% confidence intervals were calculated.

A significance level of *p* ≤ 0.05 was used in the analysis. All analyses were performed using SAS version 9.4 (SAS Institute Inc., Cary, NC).

## Results

Between July 2012 and April 2015, a total of 100 eligible patients were included. Only two patients fulfilling the eligibility criteria and approached for the study declined to participate. Median age of participants was 40 (22–75 years). Fifty one (51%) were ≤40 years. Majority (91; 91%) had infiltrating ductal carcinoma (IDC) and most patients presented with early stage disease. Eighty nine (89%) patients had positive first and/or second-degree family history of breast and/or ovarian cancers. Majority (77; 77%) of the patients had hormone-receptor (ER and/or PR) positive disease. Among the 93 patients with known HER-2 status, 13 (14%) were positive by immunohistochemistry and/or FISH (Fluorescent In Situ Hybridization). Most of the patients had grade II and III disease, Table 1.

**Table 1:** Patients characteristics, *N* = 100

Characteristics	Number (%)
Age	
Median (years)	40
Range (years)	22–75
Pathology	
IDC	91 (91%)
ILC and others	9 (9%)
Stage	
I	16 (16%)
II	51 (51%)
III	22 (22%)
IV	2 (2%)
Unknown	9 (9%)
Grade	
I	10 (10%)
II	37 (37%)
III	45 (45%)
Unknown	8 (8%)
Hormone Receptor Status	
ER and/or PR Positive	77 (77%)
ER Positive	77 (77%)
ER Negative	22 (22%)
ER Unknown	1 (1%)
PR Positive	77 (77%)
PR Negative	22 (22%)
PR Unknown	1 (1%)
HER-2 Status	
Positive	13 (13%)
Negative	80 (80%)
Unknown	7 (7%)
Triple-Negative	17 (17%)

*IDC* Infiltrating Ductal Carcinoma, *ILC* Infiltrating Lobular Carcinoma

Overall 20 (20%) patients had deleterious BRCA1 or BRCA2 mutations (7 BRCA1, 13 BRCA2). Seven (7%) patients had suspected deleterious mutations; all were in the BRCA2 gene. Seven (7.0%) variants of uncertain significance (VUS) were detected, one in BRCA1 and six in BRCA2. Table 2 summarizes the genetic and histopathologic characteristics of patients with BRCA1 and BRCA2 variants.

**Table 2:** Genetic and histopathologic characteristic of Jordanian breast cancer patients with BRCA1 and BRCA2 genetic variants

Test Result	Patients	Base change	AA change	Variant type	Interpretation	Age at diagnosis (Years)	Tumor histopathology	ER	PR	HER2	Family history (Breast/ Ovarian cancer) (n) 1st deg. 2nd deg. 3rd deg		
BRCA1 genetic variants													
3450del4	BRACA 029	del4	Stop 1115	Deletion/Frame shift	Deleterious	50	IDC/GIII	-VE	-VE	-VE	3	0	0
3954delG	BRACA 073	delG	Stop 1306	Deletion/Frame shift	Deleterious	41	IDC/GII	+VE	+VE	-VE	2	0	0
3954delG	BRACA 096	delG	Stop 1306	Deletion/Frame shift	Deleterious	37	IDC/GIII	-VE	-VE	-VE	0	1	0
3555del4	BRACA 078	del4	Stop 1153	Deletion/Frame shift	Deleterious	29	IDC/GIII	-VE	-VE	-VE	1	0	0
E1373X (4236G > T)	BRACA 091	G > T	E1373X	Nonsense	Deleterious	44	IDC/GIII	-VE	-VE	-VE	1	1	0
IVS17 + 3 A > G	BRACA 086	A > G	–	Intronic	Deleterious	34	IDC/GIII	-VE	-VE	-VE	1	1	0
5149del4	BRACA 094	del4	Stop 1678	Deletion Frame shift	Deleterious	33	IDC/GIII	-VE	-VE	-VE	1	1	0
E1478D (4553G > C)^b^	BRACA 066	G > C	E1478D	Missense	VUS	35	IDC/GII	+VE	+VE	-VE	1	2	2
E445Q (1452G > C)	BRACA 010	G > C	E445Q	Missense	FP	35	IDC/GIII	+VE	+VE	+VE	1	0	0
BRCA2 genetic variants													
999del5	BRACA 082	del5	Stop 273	Deletion/Frame shift	Deleterious	56	IDC/GIII	-VE	-VE	-VE	0	3	1
1461insA	BRACA 060 ^d^	insA	Stop 420	Insertion/Frame shift	Deleterious	33	IDC/GIII	-VE	-VE	-VE	1	2	0
1461insA	BRACA 063 ^d^	insA	Stop 420	Insertion/Frame shift	Deleterious	34	ILC/GII	+VE	+VE	-VE	1	2	0
2482del4	BRACA 018 ^d^	del4	Stop 770	Deletion/Frame shift	Deleterious	48	IDC/G UNK	+VE	+VE	-VE	2	2	2
2482del4	BRACA 070 ^d^	del4	Stop 770	Deletion/Frame shift	Deleterious	46	IDC/GII	+VE	+VE	-VE	1	2	4
2482del4	BRACA 071 ^d^	del4	Stop 770	Deletion/Frame shift	Deleterious	46	IDC/GIII	-VE	-VE	-VE	1	2	4
2482del4	BRACA 084 ^C^	del4	Stop 770	Deletion/Frame shift	Deleterious	30	IDC/GII	+VE	+VE	-VE	1	1	0
L2039X (6344 T > A)	BRACA 064	T > A	L2039X	Nonsense	Deleterious	44	IDC/GII	+VE	+VE	-VE	1	3	0
6855del8	BRACA 049	del8	Stop 2221	Deletion/Frame shift	Deleterious	33	IDC/GIII	+VE	+VE	-VE	1	2	0
6862del4	BRACA 041	del4	Stop 2227	Deletion Frame shift	Deleterious	42	IDC/GIII	+VE	+VE	-VE	2	0	0
E2229X (6913G > T)	BRACA 059	G > T	E2229X	Nonsense	Deleterious	37	IDC/GIII	-VE	-VE	-VE	1	2	1
E2229X (6913G > T)	BRACA 080	G > T	E2229X	Nonsense	Deleterious	29	IDC/GIII	+VE	+VE	-VE	1	0	0
IVS23-1G > A	BRACA 057	G > A	**–**	Intronic	Deleterious	51	IDC/GIII	+VE	+VE	-VE	3	2	0
IVS24-1G > A	BRACA 055 ^C, d^	G > A	**–**	Intronic	Suspected Deleterious	42	IDC/GIII	+VE	+VE	-VE	2	2	0
IVS24-1G > A	BRACA 061 ^C, d^	G > A	**–**	Intronic	Suspected Deleterious	55	IDC/GIII	+VE	+VE	UNK	2	2	0
IVS24-1G > A	BRACA 067	G > A	**–**	Intronic	Suspected Deleterious	32	IDC/GIII	+VE	+VE	-VE	0	2	0
dup exons 5–11(5′) ^a^	BRACA 008 ^d^	–	**–**	–	Suspected Deleterious	25	IDC/GIII	+VE	+VE	-VE	1	1	0
dup exons 5–11(5′) ^a^	BRACA 020 ^d^	–	**–**	–	Suspected Deleterious	50	IDC/GII	+VE	+VE	-VE	1	1	1
dup exons 5–11(5′) ^a^	BRACA 047	–	**–**	–	Suspected Deleterious	37	UNK	+VE	+VE	UNK	3	1	0
dup exons 5–11(5′) ^a^	BRACA 089	–	**–**	–	Suspected Deleterious	36	IDC/GIII	+VE	+VE	-VE	1	0	0
P168A (730C > G)	BRACA 093	C > G	P168A	Missense	VUS	36	IDC/GI	+VE	+VE	-VE	1	0	0
T251R (980C > G)	BRACA084 ^C^	C > G	T251R	Missense	VUS	30	IDC/GII	+VE	+VE	-VE	1	1	0
A2306P (7144G > C)	BRACA 035	G > C	A2306P	Missense	VUS	35	IDC/GII	+VE	+VE	-VE	0	1	0
Q2925R (9002A > G)	BRACA 053	A > G	Q2925R	Missense	VUS	52	IDC/GI	+VE	+VE	-VE	1	2	0
Q2925R (9002A > G)	BRACA 098	A > G	Q2925R	Missense	VUS	42	IDC/GIII	-VE	-VE	+VE	1	0	3
E2193K (6805G > A)	BRACA 099	G > A	E2193K	Missense	VUS	36	IDC/GIII	-VE	-VE	-VE	2	0	0
K21R (290A > G)	BRACA 043	A > G	K21R	Missense	FP	35	IDC/GIII	+VE	+VE	-VE	0	3	2
K3416E (10474A > G)	BRACA 055 ^C, d^	A > G	K3416E	Missense	FP	42	IDC/GIII	+VE	+VE	-VE	2	2	0
K3416E (10474A > G)	BRACA 061 ^C, d^	A > G	K3416E	Missense	FP	55	IDC/GII	+VE	+VE	UNK	2	2	0

^a^This mutation consist of a duplication of exons 5–10 of the *BRCA2* gene; the 5′ end of *BRCA2* exon 11 is also duplicated

^b^According to Myriad Genetic Laboratories- variant information sheet, this is the first observation for this variant

^C^Patients 055 & 061 had both a suspected deleterious variant and a favor polymorphism variant; patient 084 had both a deleterious variant and variant of uncertain significance

^d^The following patients are relatives: Patients 055 & 061 (sisters), patients 060 & 063 (sisters), patients 008 & 020 (second degree relatives) and patients 18, 70 & 71 (first and third degree relatives)

*AA* Amino Acid, *VUS* variants of uncertain significance, *FP* favor polymorphism, *IDC* invasive ductal carcinoma, *ILC* invasive lobular carcinoma

Excluding 5 relatives tested subsequent to the index case in their families (patients 061, 063, 020, 070 and 071), 10 (45.5%) of the 22 patients with deleterious/suspected deleterious mutations had bilateral or contralateral breast cancer, developed 2–9 years after the initial diagnosis, compared to only 5 (6.8%) out of the other 73 patients with either no known mutations, VUS or favor polymorphisms, *p*-value< 0.001, Table 3.

**Table 3:** Association of different variables with BRCA1/2 mutation status, *N*=95^a^

Variable	Level	Total	BRCA1/2 mutation status		*P*-value
			Positive (deleterious and suspected deleterious)	Others (No variant, FP, VUS)	
Age *N* = 95	age < =40	50	13(26.0%)	37(74.0%)	NS
	age > 40	45	9(20.0%)	36 (80.0%)	
Triple negative *N* = 95	No	79	13 (16.5%)	66 (83.5%)	0.001
	Yes	16	9 (56.3%)	7 (43.8%)	
Triple negative (age < =50 (*N* = 75))	No	60	12 (20.0%)	48 (80.0%)	
	Yes	15	8 (53.3%)	7 (46.7%)	0.009
Triple negative (age < =40 (*N* = 50))	No	38	7 (18.4%)	31 (81.6%)	
	Yes	12	6 (50.0%)	6 (50.0%)	0.030
Triple negative (family history = Yes (*N* = 84))	No	71	13 (18.3%)	58 (81.7%)	
	Yes	13	9 (69.2%)	4 (30.8%)	0.000
Number of relatives with breast and/or ovarian cancer (first and second degree)	Relatives < 2	31	4 (12.9%)	27 (87.1%)	NS
	Relatives > = 2	64	18 (28.1%)	46 (71.9%)	
Family history of breast cancer and/or ovarian cancer (first and second degree)	No	11		11(100%)	0.063
	Yes	84	22 (26.2%)	62 (73.8%)	
Bilateral or second primary breast cancer	no	80	12 (15.0%)	68 (85.0%)	0.000
	yes	15	10 (66.7%)	5 (33.3%)	
Family history of male breast cancer	no	90	20 (22.2%)	70 (77.8%)	NS
	yes	5	2 (40.0%)	3 (60.0%)	
Family history (age < =40 (*N* = 50))	No	10		10(100%)	0.046
	Yes	40	13 (32.5%)	27 (67.5%)	

*FP* favor polymorphism, *VUS* variant of uncertain significance, *NS* non-significant

^a^Five patients (patients 061, 063, 020, 070 and 071) were relatives to the index case tested in their families, therefore they were excluded from this analysis, see footnote to Table 2

### Young patients

Fifty one young patients (40 years or younger, range: 22–40, Median: 35 years) were included; 10 (19.6%) had deleterious mutations (4 (7.8%) BRCA1, 6 (11.8%) BRCA2). Four (7.8%) others had suspected deleterious BRCA2 mutations while 5 (9.8%) had VUS; 4 of them where in BRCA2.

Among the 40 (80.0%) young patients with positive first or second-degree family history of breast and/or ovarian cancer, 13 (32.5%) had deleterious/suspected deleterious BRCA1 or BRCA2 mutations, while no known mutations were found in the 10 other patients without a significant family history, *p*-value = 0.046. Twelve (24.0%) young patients had triple-negative disease, 6 (50.0%) had positive deleterious/suspected deleterious BRCA1/2 mutations compared to 7 (18.4%) out of the 38 none-triple negative patients (*p*-value = 0.030), Table 3.

### Triple-negative patients

Sixteen patients had triple-negative disease. Nine (56.3%) had deleterious mutations in BRCA1 or BRCA2, compared to 13 (16.5%) out of 79 patients with non-triple negative disease, p-value = 0.001 (Table 3). Six (37.5%) of these triple-negative patients had BRCA1 deleterious mutations while 3 (18.75%) had BRCA2 deleterious mutations. One triple-negative patient had a VUS in BRCA2.

### Patients with family history

Of eighty four patients (84/95; 88.4%) with first and/or second-degree family history of breast and/or ovarian cancer; 22 (26.2%) had deleterious/suspected deleterious mutations in either BRCA1 (7; 8.3%) or BRCA2 (15; 17.9%). None of the other 11 patients were positive for a deleterious/suspected deleterious mutation in BRCA 1 or 2, *p*-value = 0.063. Among the 13 patients who also had a triple-negative disease, 9 (69.2%) had deleterious BRCA1 or BRCA2 mutations, while 13 (18.3%) out of the other 71 patients who had family history but were not triple-negative harbored deleterious/suspected deleterious mutations in BRCA 1 or 2 (p-value< 0.001), Table 3.

### Other patients

Five patients had family history of male breast cancer, two (40.0%) of them had deleterious/suspected deleterious mutations in BRCA2, another patient harbored a VUS in BRCA2.

**Fig. 1:**
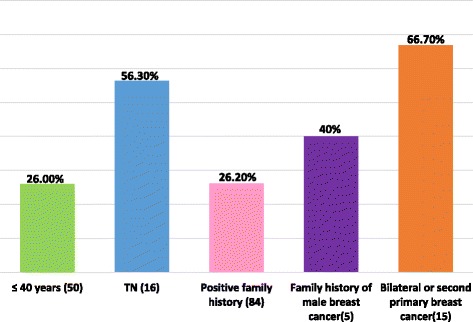
Percentage of BRCA1/2 positivity among different patients’ risk groups, *N* = 95*. Patients with BRCA1/2 deleterious or suspected deleterious mutations were considered BRCA1/2 positive. * Five patients (patients 061, 063, 020, 070 and 071) were relatives to the index case tested in their families, therefore they were excluded from this analysis, see footnote to Table 2. TN: Triple Negative breast cancer

Among the 15 patients with bilateral or second primary breast cancer; 10 (66.7%) had deleterious or suspected deleterious BRCA1/2 mutations; 5 (33.3%) were in BRCA2 and 5 (33.3) in BRCA1. Fig. 1 summarizes positive test results among different patients’ risk groups.

Using a multivariate logistic regression model, adjusting for age, triple negative status, number of first and/or second degree relatives with breast and/or ovarian cancer and bilateral or second primary breast cancer; the later three variables were significantly associated with the incidence of BRCA1/2 deleterious/suspected deleterious mutations. Odds ratios and 95% confidence intervals for triple negative, number of relatives and bilateral or second breast primary were 7.46 (1.66–33.62), 13.21 (2.20–79.30) and 19.30 (3.97–93.88), and *p*-values = 0.0089, 0.0048 and 0.0002, respectively, Table 4.

**Table 4:** Multivariate logistic regression, *N*=95^a^

Variable	Reference	OR	95% CI		*P*- value
Age	age > 40 vs age ≤ 40	0.315	0.080	1.234	0.0972
Triple negative	Yes vs No	7.460	1.655	33.624	0.0089
Number of 1st or 2nd degree relatives	Relatives ≥2 vs Relatives < 2	13.212	2.201	79.296	0.0048
Bilateral or second primary breast cancer	Yes vs No	19.304	3.969	93.882	0.0002

*OR* Odds Ratio estimates, *CI* Wald Confidence Interval

^a^Five patients (patients 061, 063, 020, 070 and 071) were relatives to the index case tested in their families, therefore they were excluded from this analysis, see footnote to Table 2

## Discussion

This is the first BRCA mutation study from Jordan. Our data showed that such mutations are not uncommon among highly selected Jordanian females with breast cancer. Using a multivariate logistic regression model, adjusting for age, triple negative status, number of first and/or second degree relatives with breast and/or ovarian cancer and bilateral or second primary breast cancer; the later three variables were significantly associated with the incidence of BRCA1/2 deleterious/suspected deleterious mutations while age was not an independent predictor of carrier status. The contribution of these findings to much younger age at diagnosis among Jordanian females is debatable. Considering the young population structure of Jordan, with around 80% of the population below the age of 40 [21], a larger fraction of breast cancer cases is expected to be younger. Nonetheless, our findings suggest that BRCA1/2 screening should be offered to patients with certain high risk features.

BRCA1/2 penetrance rates are high; results from prospective analysis of EMBRACE trial were recently reported and showed that the average cumulative risks, by age 70 years, for BRCA1 carriers were estimated to be 60% for breast cancer, 59% for ovarian cancer, and 83% for contralateral breast cancer. For BRCA2 carriers, the corresponding risks were 55% for breast cancer, 16.5% for ovarian cancer, and 62% for contralateral breast cancer [10].

Given that BRCA mutations are not uncommon and given their high penetrance rate, risk-reduction strategies including bilateral mastectomy and salpingo-oophorectomy are becoming standard of care and are widely accepted by patients and family-at-risk [22]. Most of our patients with positive deleterious/suspected deleterious mutations who were offered such risk-reduction surgeries had accepted and many already had undergone the recommended procedure(s).

International guidelines had identified specific patients with high-risk profile for which genetic counselling and testing are recommended [19, 23]. Depending on the specific ethnicity and the population studied, this group of patients can be large enough to put significant pressure on health care budgets especially in low or middle income countries, like ours, where the cost of testing is still relatively high. Identifying smaller subgroups of patients with “higher” probability of positive mutations can improve implementation of the genetic testing guidelines.

In our study, we identified subgroups of patients with significantly higher risk of having deleterious mutations. Even after excluding relatives tested subsequent to the index case in their families, 9(56.3%) patients were positive for BRCA1/2 deleterious/suspected deleterious mutations among 16 triple-negative patients. Moreover, in 12 patients with early onset triple negative breast cancer (age ≤ 40), 6 patients (50.0%) reported deleterious mutations in BRCA1/2. Such positive mutation rate was even higher (69.2%) among the 13 triple negative patients with positive first and/or second-degree family history of breast and/or ovarian cancers. The association of BRCA1 mutations with triple-negative breast cancer is well-described [24] and in our study 6 out of the 10 deleterious mutations in this subgroup were in BRCA1, Table 2.

An interesting spectrum of mutations were identified in both BRCA1 and BRCA2, Table 2. Of note, there were many recurrent mutations with more than one carrier found to harbor the identical BRCA1 or BRCA2 mutation. However, most of these carriers were either first or second-degree relatives (see footnote to Table 2) rendering this an expected finding. The small sample size of this pilot study and the fact that genetic analysis was performed at Myriad Genetics laboratories did not allow for haplotype and founder mutation analyses which will be sought in future studies. Most of the detected mutations were reported previously in the Breast Cancer Information Core (BIC) [25] among Caucasian and Western populations, possibly due to similarity of genetic makeup between Middle Eastern population and Western population [26]. Only one variant of uncertain significance (VUS) in BRCA1, E1478D (4553G > C), was reported by Myriad Genetics variant information sheet as the first observation for this variant (personal communication). The 3450del4 deleterious mutation in BRCA1 was also previously reported in patients from Egypt [25] and Tunisia [27]. Also, BRCA1 E1373X (4236G > T) was originally described in a Palestinian family [28] and was recently reported again in a Palestinian patient [29]. Similarly, the 2482del4 deleterious mutation in BRCA2 was reported among Palestinian Arabs in BIC, and the BRCA2 E2229X seems to be recurrent among Arabs [25]. It is not unexpected to find BRCA1/2 mutations among Jordanians that were previously reported in Palestinians, knowing the Palestinian-Jordanian blended nature of families in Jordan. The BRCA2 VUS Q2925R (9002A > G) was also reported in Near Eastern and Middle Eastern populations [25]. Interestingly, the Icelandic founder mutation, BRCA2 999del5 [30], was also detected in one of our patients, but we do not have data to explain this finding.

The mutation rates we are reporting are similar to what Fostira et al. had reported among 403 Greek triple-negative patients; BRCA1 mutation was found in 47.6% among a subgroup of 105 triple-negative patients with family history of breast or ovarian cancers. A rate of 35.9% was reported among a subgroup of 106 women with early-onset (< 40 years) triple-negative breast cancer [31].

In a recent study, researchers at MD Anderson Cancer Center (MDACC) reported a similar incidence of BRCA1/2 mutations in patients with ER low-positive/PR low-positive/HER-2 neu negative tumors and patients with triple-negative breast cancer, suggesting that genetic counseling and BRCA testing should also be offered to patients who have hormone receptor–low-positive breast cancers [32]. Moreover, in an earlier publication Gonzalez-Angulo et al. reported a 19.5% incidence rate of BRCA mutations among an unselected cohort of triple negative breast cancer patients and patients with mutations had a significantly lower risk of relapse [33].

Our results support the conclusion that our ethnic group is not different and as such, women with early-onset triple-negative breast cancer, and ideally all triple-negative breast cancer patients, are candidates for BRCA genetic testing especially if they have family history of breast and/or ovarian cancers.

Among the other patients’ risk groups recruited to the study, 2 out of five (40.0%) patients with family history of male breast cancer and 10 out of 15 (66.7%) patients with bilateral or second primary breast cancer reported deleterious/suspected deleterious BRCA1/2 mutations (Fig. 1). Therefore, if cost is an issue for full adaption and implementation of international guidelines in low and middle-income countries, then testing patients with these “higher” risk features can be an option, at least in the initial phases of adaptations. Since recurrent mutations in our cohort occurred mostly among first and second-degree relatives, then initial testing for these recurrent mutations cannot be recommended for cost-saving before large-scale sequencing analyses are pursed to determine BRCA1/2 mutation status. Future larger studies aiming on haplotype and founder mutation detection may help in this regard.

Our positive rates, however, are significantly higher than what have been recently reported among neighboring Lebanese women where deleterious BRCA mutations were found in only 5.6%, and an additional 12.4% with VUS [12]. The difference in mutation rates may be explained by the different testing methodology, but more importantly this difference can be justified by the different inclusion criteria. However, when comparing similar groups of enrolled patients, significant differences were still observed. Among a subgroup of 148 young Lebanese patients (≤ 40 years at diagnosis) only 9 (6.1%) harbored deleterious mutations [12], while in our cohort of 50 young patients 26.0% reported deleterious/suspected deleterious mutations. Additionally, our rate was significantly higher (32.5%) in the 40 tested young patients with positive family history compared to 10.8% in 74 similar Lebanese patients. Such differences in closely related ethnic groups are difficult to explain, but the highly selective criteria we used to include patients may still be a confounder since many of our patients satisfied more than one inclusion criterion. In addition, differences in the methodology and techniques used in BRCA testing might be a contributing factor. Moreover, considering the small number of highly selected patients included in our study, the reported BRCA1/2 mutation rates should be interpreted with caution and within context.

Conducting a culturally sensitive genetic testing research in a developing country with limited resources is a challenge. Many ethical and cultural difficulties were encountered during the course of our study. Ensuring confidentiality and privacy were major issues in a tribal-based closely-related community and culture like the Jordanian population. Many patients expressed their concerns about labeling and stigmatization. Preserving other family members’ confidentiality when documenting family history was also addressed with the patients and occasionally with the relatives. Many concerns were related to the scope of physician-patient confidentiality when relatives are at genetic risk of cancer. Sharing information with at-risk relatives was not an issue despite our IRB concerns. Except for very few (3 patients), all our patients with deleterious/suspected deleterious mutations shared results with their at-risk relatives without major issues.

Potential insurance, employment and social discrimination were also addressed with the patients prior to testing and in more detail after receiving positive mutation results. These issues are expected to be a challenge once genetic testing is made routinely available to eligible patients as a standard clinical practice, especially that most insurance agencies don’t cover risk-reduction procedures including contralateral mastectomies and oophorectomies.

Following this exploratory pilot study, BRCA testing has started to be routinely offered at our institution, initially for the “higher” risk groups (discussed above) with the intention to gradually expand to include a wider patient population as suggested by the ASCO (American Society of Clinical Oncology) [23] and the NCCN (National Comprehensive Cancer Network) guidelines [19]. This process should enhance our understanding of the prevalence of BRCA1/2 mutations in our patient population. An ongoing project is currently collecting this information prospectively on all patients tested for BRCA1/2 mutations. The results of this project will help to assess whether a founder effect exists in the Jordanian population and whether a subset of mutations can be tested for cost-saving. Future recommendations for establishing a Clinical Cancer Genetics program are envisioned, where unaffected family members can also benefit from early screening and take appropriate risk-reduction measures.

Our study is limited by the small sample size and the highly selective criteria used for patient accrual. We were conscious to these limitations from the onset of the study. The small sample size was due to the limited funds available and to the high cost of BRCA testing at an accredited and reliable laboratory. Therefore we opted for highly selective inclusion criteria to test the more high risk patients in order for the results to have relevance in the clinical setting, especially that colleagues at other academic institutions in Jordan were reporting lack of BRCA1/2 mutations among Jordanians based on scholarly research performed in-house in their laboratories (personal communication). Our selected patient cohort shows an important incidence of deleterious and suspected deleterious BRCA mutations suggesting that genetic testing should be discussed with and offered to patients with such a high risk profile. Further studies are needed to confirm the results of this pilot study. Moreover, since many of the recruited high risk patients tested negative for BRCA1/2 mutations, it is plausible to take advantage of the collected DNA samples and test for mutations in other breast cancer susceptibility genes, e.g. CHEK2, PALB2 and BRIP1. Using next-generation sequencing will enable simultaneous testing for mutations in these and other genes, and multigene panels are now commercially available and are increasingly being used [34–36].

## Conclusions

In summary, our results support the conclusion that BRCA1/2 mutations are common among Jordanian breast cancer patients with a highly selected risk profile and may contribute to the pathogenesis of disease in this patient population. This has significant clinical implications, both for the management and prevention of breast cancer. Therefore, full BRCA1/2 screening should be offered to patients with characteristic high risk features.

## Abbreviations

ASCO: American Society of Clinical Oncology
BIC: Breast cancer Information Core
BRCA1: BReast CAncer susceptibility gene 1
BRCA2: BReast CAncer susceptibility gene 2
ER: Estrogen Receptor
FISH: Fluorescent In Situ Hybridization
FP: Favor Polymorphism
HER2: Human Epidermal growth factor Receptor 2
IDC: Infiltrating Ductal Carcinoma
ILC: Infiltrating Lobular Carcinoma
IRB: Institutional Review Board
NCCN: National Comprehensive Cancer Network
PCR: Polymerase Chain Reaction
PR: Progesterone Receptor
SINF: Sister Institution Network Fund
VUS: Variant of Uncertain Significance
